# Automatic generation of learning path for teaching informatics at university

**DOI:** 10.1016/j.mex.2025.103634

**Published:** 2025-09-17

**Authors:** Aynur Aliyeva, Ali Abbasov

**Affiliations:** Ministry of Science and Education of Institute of Control Systems, Baku, Azerbaijan

**Keywords:** Adaptive learning system, Concept map, Informatics, K-means

## Abstract

The article discusses the problem concept maps are crucial tools for visualizing informatics knowledge in adaptive learning systems. In computer science education, concept maps are essential for visualizing complex knowledge structures and supporting adaptive learning. This study presents a learning path generation (LPG) algorithm designed to improve personalized learning in computer science. By analyzing students’ concept maps, the LPG algorithm effectively distinguishes between groups of students based on their performance and generates customized learning paths.

The LPG algorithm uses advanced computational methods to analyze concept maps and learning paths. Then, simplified learning paths are generated using a topological sorting algorithm. By using this method in computer science education, the following objectives can be achieved:

Effectively distinguish between groups of students and generate customized learning paths based on their performance.

Knowledge acquisition and increased student engagement.

Learning path generation can be applied to any subject area.

## Graphical abstract

**Figure:**
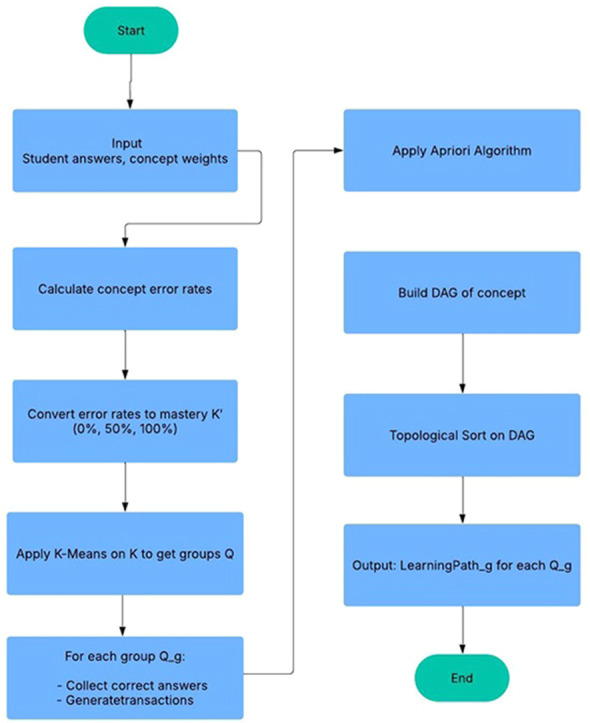

Specifications table

**Table:** 

**Subject area**	Computer Science
**More specific subject area**	Informatics
**Name of your method**	Lee et al.’s method (2009)
**Name and reference of original method**	Yancong Lı, Zengzhen Shao, Xıao Wang, Xuechen Zhao and Yanhuı Guo, A concept map-based learning paths automatic generation algorithm for adaptive learning systems. IEEE Access. VOLUME 7, 2019. pp. 245–255. doi: https://doi.org/10.1109/ACCESS.2018.2885339
**Resource availability**	https://gist.github.com/nuray2010/9e783142ac3dab9a31dfc18a5d7d5251

## Background

The teaching of the informatics subject in adaptive systems holds great significance for the development and application of modern technologies. Acquiring deep knowledge in the field of informatics and creating and implementing optimal solutions in dynamic and complex environments requires teaching tailored to the individual needs of students in adaptive learning systems. Adaptive systems analyze the learning abilities and interests of students to create personalized learning plans.

They offer suitable resources and teaching methods for each student, monitor their progress, and identify weaknesses. Adaptive testing methods are applied to accurately assess the student's knowledge level. The content of the subject is directed toward topics aligned with the student's knowledge needs. Traditional education systems are often based on standardized approaches, which may not be effective for learners with diverse skills and interests. Adaptive learning, with its personalized teaching approaches, creates a flexible education system that meets the demands of modern times, enabling the application of the most suitable teaching methods for everyone. This ensures more effective learning [[1], [2], [3]].

However, to guarantee the efficiency and effectiveness of these systems, complex mechanisms are required to structure and guide teaching materials [[4], [5], [6]]. Concept maps emerge as a useful tool in this context, offering a visual representation of knowledge domains by organizing concepts and their interrelations [7,8]. They allow learners to understand complex subjects in a clear and intuitive way while enabling teachers to design more structured and coherent curricula. The integration of concept maps into adaptive learning systems can facilitate the creation of automatic learning paths by aligning educational content with the learner's existing knowledge base and learning objectives.

This article presents the application of an algorithm for the automatic creation of concept maps in the teaching of informatics. The use of concept maps in adaptive learning systems in higher education to improve the quality of informatics teaching is a relevant issue, making concept maps widely used in adaptive teaching systems.

***The Theory of Concept Maps.*** For the first time in the 1970s, Novak and Gowin [9,10] conducted research at Cornell University on the visualization of knowledge in subjects and proposed concept maps to illustrate the relationships between concepts (topics) of the subject. Their main goal was to apply David Ausubel's Assimilation Theory to measure students' knowledge and understanding. The primary purpose of concept maps is to structure and visualize knowledge and concepts. They aim to show relationships between topics, make information easier to understand and remember, simplify teaching and learning processes, identify topics that students struggle to master, and help them focus on those areas.

In the 1990s, with advancements in artificial intelligence and data analysis, interest in the automatic creation of concept maps began to grow. Tools for Natural Language Processing (NLP) were developed for text analysis. Initial studies focused on extracting key concepts and building relationships from texts. Statistical models were used to detect concepts frequently occurring together in texts [11,12].

In the 2000s, ontologies (such as OWL and RDF technologies) and semantic networks played a significant role in structuring data and automating the creation of concept maps [13,14]. In the 2010s, artificial intelligence and machine learning began to advance further. Machine learning algorithms enabled more precise extraction of concepts and relationships from data. During this period, numerous algorithms and applications were developed for creating automatic concept maps. Transformer models (e.g., BERT, GPT) made it possible to understand relationships in texts more deeply, leading to more accurate creation of automatic concept maps [15,16].

It is worth noting that in the 2020s, the extraction of knowledge from big data and the creation of network graphs became popular. Artificial intelligence models began to be widely used for the automatic analysis of data, identification of concepts, and visualization of relationships [[17], [18], [19], [20]].

***The Construction of Concept Maps.*** The construction of concept maps utilizes two cognitive theories of memory: Ausubel’s Assimilation Theory [1968] and Disan’s Associative Theory [1965].

According to the Assimilation Theory, memory is hierarchical, and new information is processed and stored as either a more general or more specific concept relative to other related concepts, meaning it is assimilated into the existing structure [Fraser, 1993].

The Associative Theory suggests that while memory supports hierarchies, it consists of a network of non-hierarchical concepts. When two concepts overlap to some extent, relationships naturally emerge between them. The memory structure described by the Associative Theory closely resembles that of the Assimilation Theory, except that hierarchies are not required.

The method for developing concept maps depends on which of these two theories is applied and is explained in more detail below. A concept map is a graph composed of nodes connected by edges. The edges in the graph indicate that the concepts are conceptually and logically related in a certain way.

Novak provided the following guidelines for constructing concept maps. Concepts and keywords may vary depending on the lecture topic. Maps pre-prepared by experts (teachers) can be presented to students as teaching tools. When using concept maps to deliver knowledge, they ensure a sequence that better aligns with structured teaching objectives and pedagogy while providing more information than a standard outline about the relationships between concepts.

Concept maps offer a visual representation of conceptual and interconnected knowledge within a particular domain. The graph is composed of nodes, representing concepts, and edges that illustrate the relationships between them. As students learn, they can create alternative conceptual maps by making associations, rather than taking traditional notes. Teachers can predefine the graph’s nodes (concepts) and their relationships for students.

Concept maps also help teachers evaluate how students relate the material they understand to the overall objectives of the course.

## Method details

The automatic construction of concept maps in education offers significant benefits and plays a crucial role in improving the quality of the teaching process, visualizing students’ knowledge, and creating personalized learning environments. Automatic concept maps present educational topics and the relationships between those topics in a clearer and more structured manner. Students gain deeper knowledge by understanding the connections between topics. Breaking down complex subjects into sub-concepts and illustrating their relationships makes learning easier.

Systems that generate automatic concept maps can create personalized concept maps tailored to the student’s level of knowledge. For instance, simplified maps can be generated for students with lower levels of knowledge, while more complex maps are designed for those with higher levels of understanding. These systems can automatically identify gaps in the student’s knowledge and create concept maps that focus on those areas. Teachers, taking into account students' levels of comprehension, can devise more effective teaching strategies. Areas where students face difficulties can be automatically analyzed, enabling teachers to build more efficient lesson plans based on the analysis of concept maps. Automatic concept map generation systems save time for both teachers and students. These systems represent a significant step forward in the development of educational technologies.

This article examines the issue of constructing automatic concept maps in higher education for the teaching of informatics by grouping students based on their learning levels and using data mining methods to generate concept maps tailored to each group.

Suppose n students $L_{1},\;L_{2},\;\ldots \;,\;L_{n}\;$ have answered m test questions $T_{1},\;T_{2},\;\ldots \;,\;T_{m}$. Then the m test questions are associated with j concepts. A weight matrix between the test questions and the concepts is given. The weights of the concepts $C_{i},\;i=1,2,\ldots ,j$ for those test questions are determined by the teacher. Since a question belongs to multiple concepts, the student features are derived based on the error rate of each student's answers for each concept. That is, for each student, the features $K=\;\{k_{1},\;k_{2},\;\ldots ,\;k_{j}\}$ representing their mastery of each concept are calculated based on their answers across all questions. Here, j is the number of concepts, and $k_{j}\in \left[0,1\right],$ represents the error rate of the student's answers for concept j. The higher the value of $k_{j}$, the lower the student's mastery of the j concept.

**Fig. 1:**
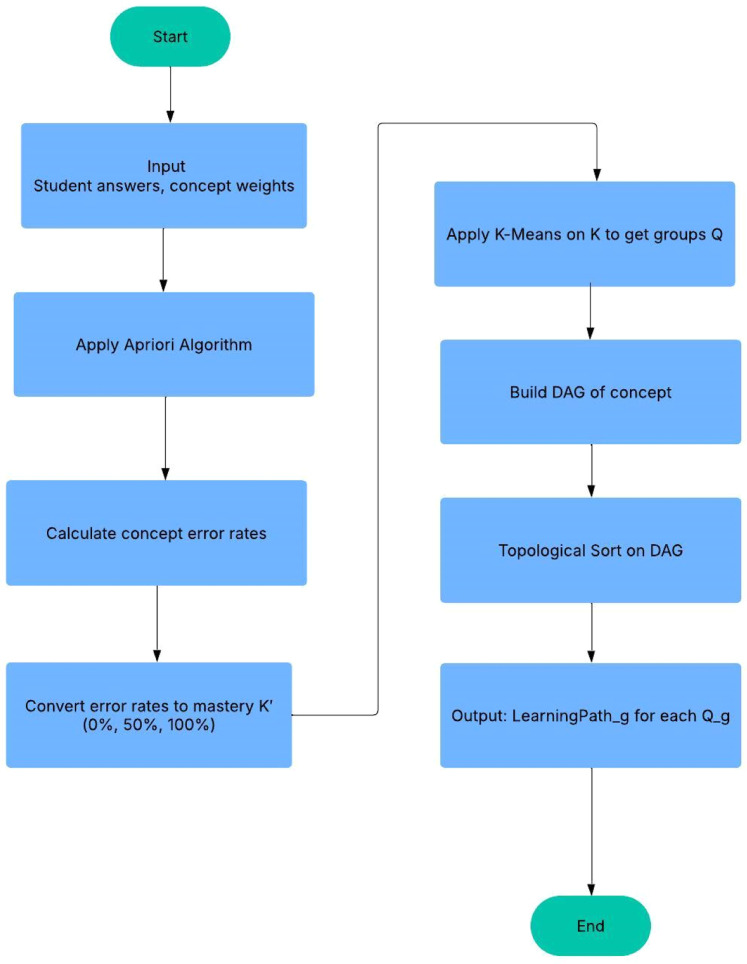
The flow chart of the LPG algorithm.

Based on the error rate of each concept for a student, $K^{\prime }=\;\left\{k_{1}^{{}^{\prime }},\;k_{2}^{{}^{\prime }},\;\ldots ,\;k_{j}^{{}^{\prime }}\right\},$ student features can be converted into mastery levels, where $\;k_{j}^{{}^{\prime }}=\;\left\{0\%,\;\;50\%,\;\;100\%\right\}$. The mastery level can be categorized as low level, medium level, and high level. The flow chart of the Learning Path Generation algorithm is shown in Fig. 1 and Table 1.

**Table 1:** Pseudocode LPG algorithm.

Inputs:
- R: Students' test records
- QC: Relationships between questions and concepts
For each student feature $k_{j}\in K$:
- Analyze performance on each concept c using R and QC.
- Compute error rates per concept.
For each concept c and each student:
If error rate > 66 % → Mastery = 0 %
If 33 % < error rate ≤ 66 % → Mastery = 50 %
If error rate ≤ 33 % → Mastery = 100 %
Form new feature vectors $K^{\prime }=\;\left\{k_{1}^{{}^{\prime }},\;k_{2}^{{}^{\prime }},\;\ldots ,\;k_{j}^{{}^{\prime }}\right\}$ representing mastery.
Apply K-means clustering on $K^{\prime }$:
- Input: Student mastery features $K^{\prime }$
- Output: i clusters $Q=\;\{Q_{1},\;Q_{2},\;\ldots ,\;Q_{i}\}$
Using frequent concept sets (from Apriori) and mastery levels:
- Automatically produce a concept map.
- Build a Directed Acyclic Graph (DAG) to represent this structure.
Each Gᵢ is a group of students with similar learning needs.
Perform Topological Sort on the concept DAG:
- This gives a valid learning order where prerequisites precede dependents.
Output:

Each student group receives a personalized learning path based on their mastery levels and concept dependencies.

### Cluster analysis of students

**Fig. 2:**
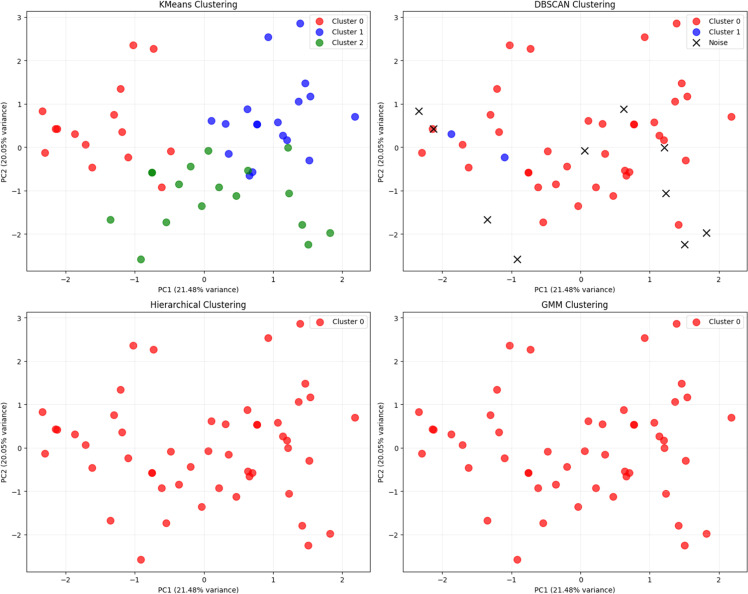
Clustering methods.

After determining the students' level of comprehension, clustering is performed based on their learning abilities. For this purpose, the K-Means algorithm is applied. The K-Means algorithm is a classical method in cluster analysis [21]. In the K-Means algorithm, the number of clusters must be specified prior to clustering. In this study, multiple clustering algorithms were applied to group students based on their mastery of educational concepts, with the goal of generating personalized learning paths. To ensure methodological rigor, we conducted a comparative analysis of K-means, DBSCAN, Hierarchical Clustering, and Gaussian Mixture Models (GMM), and evaluated their performance using standard internal clustering validation metrics—namely, Silhouette Score and Davies–Bouldin Index (DBI). To identify meaningful student groupings based on their concept mastery and response patterns, we applied multiple clustering algorithms and evaluated their performance using standard internal validation metrics: Silhouette Score and Davies–Bouldin Index (DBI). Additionally, we considered the number of clusters generated and the proportion of data marked as noise. In addition, Table 2 and Fig. 2 illustrate comparison of clustering methods.

**Table 2:** Comparison of clustering methods.

Method	Silhouette Score	Davies–Bouldin Index	Number of Clusters	Noise Points
K-means	0.1487	1.8586	3	0
DBSCAN	–1.0000	∞ (undefined)	2	10

As can be seen from the table K-means produced three clusters with a positive silhouette score (0.1487) and an acceptable DBI (1.8586), indicating moderate but usable separation between student groups. All data points were assigned to a cluster, ensuring complete student coverage.

On the other hand DBSCAN, performed poorly, with a negative silhouette score (–1.0) and an undefined DBI, which reflects poor cluster cohesion and separation. Furthermore, 10 students (20 % of the dataset) were marked as noise, rendering the results less useful for educational interventions.

The Hierarchical Clustering method likely grouped all data into a single cluster or failed to produce a meaningful partition. This often occurs if the data lacks strong ierarchical structure.

Gaussian Mixture Model (GMM) to a single Gaussian component or could not distinguish between distributions. This often happens when the data is binary or not normally distributed — which is common in educational datasets involving right/wrong answers.

The selection of K-means is thus not arbitrary but grounded in both empirical results and the specific characteristics of the dataset. Educational response data typically consist of categorical or binary outcomes (e.g., correct/incorrect answers), and K-means is well-suited to this structure when the goal is to produce clear, mutually exclusive student profiles. Furthermore, its simplicity, scalability, and interpretability make it highly applicable in adaptive learning environments, where timely and actionable groupings are essential.

Therefore, K-means was selected as the primary clustering technique for generating personalized learning paths. Its performance and suitability support its use in educational data mining contexts, particularly when working with concept-level mastery data in systems that aim to deliver adaptive instruction.

Students can be divided into several groups based on their comprehension abilities. Each group can be analyzed separately. By applying K-Means clustering to the students' answers in the Informatics subject, their answers are analyzed and grouped. After grouping students based on their level of comprehension, the groups are denoted as $Q=\;\{Q_{1},\;Q_{2},\;\ldots ,\;Q_{i}\}$, where i represents the number of groups.

### Approach for the automated generation of concept maps

Later, for each group, a concept map is created based on the students' answers to test questions using association rules. Suppose that n students gave$\;L_{1},\;L_{2},\;\ldots \;,\;L_{n}$ answers to $\;T_{1},\;T_{2},\;\ldots \;,\;T_{m}$ m test questions. Then the scoring matrix G is given as follows:

$$G=\left[\begin{array}{ccc} g_{11} & \cdots & g_{1m}\\ \vdots & \ddots & \vdots \\ g_{m1} & \cdots & g_{mn} \end{array}\right],$$

where $g_{ij}\epsilon \left[0,1\right]$ indicates the incorrect answer of student $L_{j}$ to test question $T_{i}\;$as $g_{ij}=0$, and the correct answer of student $L_{j}$ to test question $T_{i}\;$as $g_{ij}=1$, i=1,2,…,m,j=1,2,…,n. The weight matrix between test questions and concepts will be as follows:

$$TC=\left[\begin{array}{ccc} tc_{11} & \cdots & tc_{1n}\\ \vdots & \ddots & \vdots \\ tc_{m1} & \cdots & tc_{mp} \end{array}\right],$$

where $tc_{ij}$ denotes the weight of the concept $C_{j}$ to which the question $T_{i}$ belongs, $0\leq tc_{ij}\leq 1,\;\;i=1,2,\ldots ,m,\;\;j=1,2,\ldots ,p$. The proposed method for building a concept map based on the answers to the test questions consists of the following steps.

The algorithm that determines association rules between the elements. Based on the student's answers to the test questions and the Apriori algorithm (Agrawal and Srikanth, 1994), the following association rule is determined between the questions [22]:

1. If a student answered question $T_{i}$ correctly, that person also answered question $T_{j}$ correctly.
2. If a student answered question $T_{i}$ incorrectly, that person also answered question $T_{j}$ incorrectly.

For each question pair, we create association rules based on students correct and incorrect answers for each question in that pair and calculate their support value. Then, new question sets are created, each consisting of two questions. We denote these question sets by $\left(T_{i}\to T_{j}\right)$. For each question pair $sup(T_{i}\to T_{j}),$i≠j,i=1,2,…,m,j=1,2,…,m,I confidence value is calculated:

(1) $$I\left(T_{i}\to T_{j}\right)=\frac{sup\left(T_{i}\to T_{j}\right)}{sup\left(T_{i}\right)}$$

where, first, the support values $sup(T_{i})\;$are calculated for all questions $T_{i}\;i=1,2,\ldots ,m$, one by one, and the questions that satisfy the minimum support are selected. Assume that questions have a minimum support value of 40 %, that is, 1-element answers must have a support value of at least two. We calculate the degree of relationship between concepts in question pairs based on the concept weight assigned by the teacher for each question. If there is more than one relationship between any two concepts, then we take the one with the highest relationship degree.

For questions with correct and incorrect answers, we calculate the relationship between concepts, using the following formula

(2) $$\begin{array}{c} R{\left(C_{i}\to C_{j}\right)}_{T_{x}T_{y}}=\min\left(W_{T_{x}C_{i}},\;W_{T_{y}C_{j}}\right)\times I\left(T_{i}\to T_{j}\right), \end{array}$$

where $R{\left(C_{i}\to C_{j}\right)}_{Q_{x}Q_{y}}\in \left[0,1\right]$ denotes the degree of relationship between the concepts $"C_{i}\to C_{j}"\;$and the questions $T_{x}\to T_{y}$, i=1,2,…,p,j=1,2,…,p,x=1,2,…,m,y=1,2,…,m. Here, $W_{T_{x}C_{i}}\;$ denotes the weight of the concept $C_{i}\;$in the question$\;T_{x}$, $W_{T_{y}C_{j}}\;$the weight of the concept $C_{j}$ in the question $T_{y}$.

After finding connections, we combine the relationship degrees for correct and incorrect answers for concepts $C_{i}$ and $C_{j}$ in one table, satisfying the following conditions:

• If there is a relationship in the “$C_{i}\to C_{j}$” relationship table, we write it in the combined relationship table.
• If there are two relationships in the “$C_{i}\to C_{j}$” relationship table, we calculate the relationship difference with the following formula and write it in the combined relationship table:
  (3) $$R{}^{\prime }=\frac{R{\left(C_{i}\to C_{j}\right)}^{+}-\;R{\left(C_{i}\to C_{j}\right)}^{-}}{MAX(R{\left(C_{i}\to C_{j}\right)}^{+},\;\;\;R{\left(C_{i}\to C_{j}\right)}^{-}},$$

where $R{\left(C_{i}\to C_{j}\right)}^{+}$ is the degree of conceptual relationship of correct answers, $R{\left(C_{i}\to C_{j}\right)}^{-}$i=1,2,…,p,j=1,2,…,p, is the degree of conceptual relationship of incorrect answers. The table is constructed within the following conditions that the quantity $R{}^{\prime }$ satisfies:

• If the difference is greater than a certain number μ,0≤μ≤1 set by the user, we delete the relationship between the concepts $C_{i}$ and $C_{j}$. Otherwise, we write the one with the largest degree of correspondence between the concepts $C_{i}$ and $C_{j}$ into the relationship table.
• If the confidence level between concepts $C_{i}$ and $C_{j}$ is smaller than the number set by the user, we delete it.

After the concept maps are constructed for each group, the sequence of concepts is presented to the student. This is because the generated graphs depict which topic should be studied before the other. However, this does not allow the student to efficiently use the graph. Since the concept map is a directed acyclic graph (DAG), we apply a topological sorting algorithm. After applying the topological sorting algorithm, we generate the learning path for each group.

## Method validation

The experiment was conducted based on the answers of 20 students to 15 test questions covering 5 concepts of the subject Informatics, as shown in Table 3 and Table 4. The proposed algorithm, when applied during the semester in higher education institutions, will help identify topics that students have not learned and assist in learning them in a more efficient way.

**Table 3:** Students' answers to questions.

	T1	T2	T3	T4	T5	T6	T7	T8	T9	T10	T11	T12	T13	T14	T15
L1	0	1	1	1	1	0	0	1	0	1	1	0	1	0	0
L2	0	0	0	0	1	0	0	1	1	0	1	0	1	1	1
L3	1	0	1	0	1	1	0	1	0	0	0	1	1	0	1
L4	0	0	1	1	0	0	0	1	0	1	0	1	0	0	1
L5	0	0	0	1	0	1	0	1	1	1	1	0	0	0	1
L6	0	1	0	1	1	0	0	0	0	1	1	1	0	1	1
L7	0	0	0	1	1	1	1	0	1	1	0	1	1	1	1
L8	1	1	0	1	1	1	1	1	0	0	1	1	0	0	1
L9	1	0	1	0	1	0	1	0	0	1	0	0	0	0	0
L10	1	1	1	1	1	1	1	0	1	1	1	0	0	1	1
L11	0	1	0	1	1	1	0	0	1	1	1	0	1	1	1
L12	1	1	1	1	1	0	0	0	1	0	0	1	1	1	0
L13	0	0	0	0	0	0	1	0	1	1	0	1	0	1	1
L14	1	0	1	0	1	0	0	0	0	0	0	1	0	0	0
L15	1	0	1	1	1	1	1	0	0	1	1	1	0	1	0
L16	1	0	0	0	0	0	0	1	0	1	1	0	0	1	0
L17	0	1	0	1	1	1	0	1	1	0	1	0	0	0	0
L18	0	0	0	0	0	1	0	1	1	0	1	1	0	0	1
L19	0	1	1	0	0	0	0	1	0	1	1	0	0	1	0
L20	1	1	1	0	1	1	0	1	1	1	1	1	1	1	0

**Table 4:** Question-concept relationship.

	C1	C2	C3	C4	C5
T1	0.35	0.33	0.0	0.0	0.32
T2	0.0	0.0	0.0	0.0	1.0
T3	0.46	0.54	0.0	0.0	0.0
T4	0.0	1.0	0.0	0.0	0.0
T5	0.0	0.0	0.0	1.0	0.0
T6	0.35	0.0	0.0	0.0	0.65
T7	0.36	0.0	0.0	0.64	0.0
T8	0.0	0.14	0.0	0.42	0.45
T9	0.31	0.69	0.0	0.0	0.0
T10	0.0	0.0	1.0	0.0	0.0
T11	0.1	0.0	0.0	0.9	0.0
T12	0.0	0.0	0.0	1.0	0.0
T13	0.64	0.19	0.17	0.0	0.0
T14	0.0	0.81	0.17	0.02	0.0
T15	1.0	0.0	0.0	0.0	0.0

The number of concepts between 4 and 7 allows for the construction of a more effective concept map. It should be noted that having too many concepts makes analysis difficult. The relationship between concepts and questions is determined by experts (teachers). The sum of the concept weights for the questions is one unit. For all students, based on their answers to all questions, features reflecting the mastery of each concept are identified. In other words, for each concept, based on the student's incorrect answer rate, student features are transformed into a mastery level. The students' mastery level can be low, medium, or high.

Since the number of students in the group is 20, we have taken the number of clusters as 3. Therefore, after clustering into three groups, we have obtained the following groups. After executing the algorithm, it offers different learning paths for each group.

**Fig. 3:**
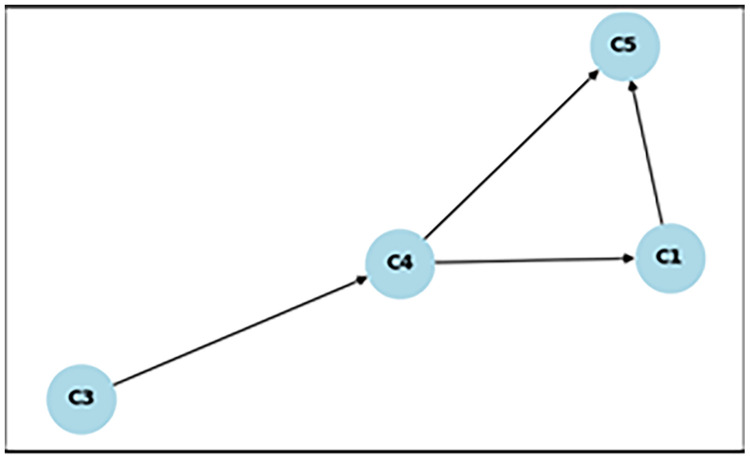
Concept map all groups.

Fig 3. shows the concept map calculated for the entire group. After clustering the students according to their learning level, groups are created.

As can be seen in Table 5 it is possible to calculate and sort different graphs for each group. Creating and sorting a concept map for each group allows you to present it to the students. This in turn allows you to tailor the teaching process to each student.

**Table 5:** Learning path.

Group	Student id number	Learning path
all	1,2,3,4,5,6,7,8,9,10,11,12,13,14,15,16,17,18,19,20	С3→С4→С1→С5
I	3, 8, 17	С4→С2→С1→С5→С3
II	10, 12, 20	С1→С2→С1→С2→С2
III	1, 2, 4, 5, 6, 7, 9, 11, 13, 14, 15, 16, 18, 19	С3→С2→С4→С1

## Conclusions

The development of concept maps in Informatics education has proven to be a powerful method for structuring and personalizing the learning process. This study proposed a data-driven approach for generating automatic learning paths that adapt to the unique mastery profiles of student groups. By leveraging clustering techniques, the proposed algorithm efficiently categorizes students into meaningful groups based on their knowledge gaps and competencies. Among the clustering methods tested, K-means clustering demonstrated the most reliable performance, yielding interpretable and well-separated student clusters. Evaluation metrics such as the Silhouette Score (0.1487) and Davies–Bouldin Index (1.8586) supported the use of K-means over alternatives like DBSCAN, GMM, and Hierarchical clustering, which failed to produce useful or stable groupings. This indicates that the student performance data is better captured by partition-based clustering rather than density- or distribution-based models.

Once grouped, student clusters were used to generate individualized concept maps that reflect the specific needs and strengths of each group. The Apriori algorithm was employed to identify essential topic sequences within each group’s knowledge profile. Using topological sorting, these sequences were transformed into adaptive learning paths, enabling a structured and personalized progression through course content.

We would like to highlight the following key findings from this article:

• K-means clustering is an effective strategy for partitioning students based on mastery levels in Informatics, offering clear and actionable groupings.
• The integration of concept maps with adaptive learning paths supports both diagnostic and formative educational interventions.
• Automatic concept mapping enhances transparency, facilitates personalized instruction, and helps teachers identify student needs in real-time.
• The system provides a scalable, data-informed solution for optimizing teaching strategies and individualizing content delivery.

The integration of automatic concept maps into digital learning platforms presents a promising direction for the future of education. This approach enables instructors to tailor the learning experience at scale while ensuring that students progress through material in a logical and personalized manner. As educational data becomes increasingly available, such adaptive systems will play a critical role in deepening student understanding and improving learning outcomes in Informatics and beyond.

## Limitations

Not applicable.

## Ethics statements

No

## Declaration of competing interest

The authors declare that they have no known competing financial interests or personal relationships that could have appeared to influence the work reported in this paper.

## Data Availability

The data that has been used is confidential.
